# Prevalence of Asymptomatic Hiatal Hernia in Obese Patients During Preoperative Upper Gastrointestinal Endoscopy Assessments and Correlation With Body Mass Index

**DOI:** 10.7759/cureus.13396

**Published:** 2021-02-17

**Authors:** Bandar Saad Assakran, Khaled Alrakbi, Meshari A Alharbi, Moath A Almatroudi, Asim Alshowaiman, Abdullah Homood Alromaih, Naif Alaqil, Basil Alharbi, Ali Alsoghayer

**Affiliations:** 1 General Surgery, King Fahad Specialist Hospital, Buraydah, SAU; 2 General Surgery, King Fahad Specialist Hospital, Burydah, SAU; 3 General Surgery, Unaizah College of Medicine, Unaizah, SAU; 4 General Surgery, Qassim University, Burydah, SAU

**Keywords:** hiatal hernia, obese patient, upper gastrointestinal endoscopy, bmi

## Abstract

Introduction

In obese patients, hiatal hernia (HH) can be asymptomatic or may present with one or few symptoms, such as heartburn, nausea, or vomiting. Routine upper gastrointestinal (GI) endoscopy is the most frequent method used to determine the presence of any abnormalities, including HH. This study aimed to assess the prevalence of asymptomatic HH in obese patients during routine upper GI endoscopy assessments and to examine the correlation with body mass index (BMI).

Materials and methods

This was an observational, retrospective cohort study conducted at King Fahad Specialist Hospital, Buraydah, Qassim, Saudi Arabia. The data were extracted from the medical records and electronic charts of all obese patients who underwent preoperative upper GI endoscopy assessment between January 2017 and December 2019. Data were tabulated in Microsoft Excel and analyzed using the Statistical Package for the Social Sciences (SPSS) Version 21 (IBM Corp., Armonk, NY, USA).

Results

Among the 690 obese patients, HH was found in 103 (14.9%) patients. The chi-square test revealed that abdominal pain (X^2^=3.885; p=0.049), shortness of breath (X^2^=8.057; p=0.005), vomiting (X^2^=4.302; p=0.038), nausea (X^2^=4.090; p=0.043), and other HH symptoms (X^2^=3.897, p=0.048) were the most frequently reported HH related symptoms, but BMI was not (X^2^=2.126; p=0.345). In the multivariate regression model, the use of PPI (proton-pump inhibitor) medication (adjusted OR [AOR]=0.237; 95% CI=0.074-0.760; p=0.023) was found to be higher in those with HH. Vomiting (AOR=1.722; 95% CI=1.025-2.890; p=0.040) and nausea (AOR=1.698; 95% CI=1.012-2.849; p=0.045) were the most frequently reported symptoms related to HH.

Conclusion

Asymptomatic HH among obese patients is not widely prevalent in our region. The use of PPI medications was found to decrease the symptoms associated with HH, such as vomiting and nausea. However, there was no evidence linking BMI to the development of HH.

## Introduction

Obesity can affect one’s health in many ways, including the incidence of hiatal hernia (HH), that is, dilation or weakness of the diaphragmatic opening through which the esophagus passes. This dilation can cause a part or the entirety of the stomach to migrate into the thoracic cavity [1]. A prospective study conducted in the University of Alabama at Birmingham Hospital, Birmingham, AL, USA, included 1,224 participants who underwent upper gastrointestinal (GI) endoscopy and found that 65% of patients with an increased waist-to-hip ratio presented with esophagitis or HH [2]. Patients with HH or esophagitis can present with few or no symptoms. It can be found incidentally while investigating digestive disorders using upper GI tract endoscopy [3].

According to Hill’s classification, HH is classified based on endoscopic findings into the following: sliding HH, which is the most common type (95% of patients); para-esophageal HH, which is seen when the lower esophageal sphincter remains preserved while the fundus of the stomach herniates through the diaphragm; mixed type; and the fourth type, which involves migration of the stomach or bowel. The common symptoms of this disease include dysphagia, heartburn, regurgitation, nausea/vomiting, chest pain, and abdominal pain [4,5]. There are multiple risk factors associated with HHs, including age, sex, race, body mass index (BMI), or any increase in intra-abdominal pressure [1]. HHs can be detected with multiple techniques. However, only two techniques can accurately diagnose HHs: barium swallow and upper endoscopy [4].

Considering the variations in the incidence and frequency of HH among obese patients and the correlation with asymptomatic HH between studies and considering that the prevalence of asymptomatic HH in the Al-Qassim province in Saudi Arabia has not yet been established, we conducted a retrospective study on this topic. We then compared the results with those of other studies conducted in and outside Saudi Arabia to fully understand its prevalence. This study aimed to determine the prevalence of asymptomatic HH in obese patients during routine upper GI endoscopy preoperative assessment and to assess the relationship between BMI and the presence of HH. This article was previously posted to the Research Square preprint server on October 23, 2020 [6].

## Materials and methods

An observational retrospective cohort study was conducted at King Fahad Specialist Hospital, Buraydah, Qassim, Saudi Arabia. The study was approved by the Institutional Review Board of the National Bioethics Committee in the Qassim province. The data were extracted from the medical records and ambulatory records of all obese patients (BMI > 30) who underwent preoperative upper GI endoscopy assessment between January 2017 and December 2019. Demographic, clinical, and endoscopic data were collected from electronic health records.

Qualitative data were expressed as frequencies and percentages, and quantitative data were expressed as the mean and standard deviation. The relationship between HH and the basic demographic characteristics and associated diseases of obese patients was established using a chi-square test. A non-parametric test was used for non-normally distributed variables, and the variables were expressed as medians and interquartile ranges. A multivariate regression analysis was also performed to determine the independent significant factors associated with HH, where the adjusted ratio and 95% confidence interval were also reported. A p-value of <0.05 was considered statistically significant. All statistical analyses were performed using the Statistical Package for the Social Sciences (SPSS) Version 21 (IBM Corp., Armonk, NY, USA).

## Results

Data of 690 obese patients who underwent preoperative upper GI assessment between January 2017 and December 2019 were analyzed. Table 1 presents the basic demographic characteristics of the patients. The patients’ ages ranged from 15 to 63 years (mean: 33.9 years), with 26-35 years being the most common age group (33.9%). Females (57.1%) were slightly more prevalent than males (42.9%). Furthermore, nearly all patients were Saudis (99.4%). More than half (54.1%) of the patients were classified as morbidly obese. In addition, only 1.7% of patients used PPI medications.

**Table 1:** Basic demographic data of obese patients (n=690) BMI, body mass index; PPI, proton-pump inhibitors

Study Data	N (%)
Age group	
15–25 years	182 (26.4%)
26–35 years	234 (33.9%)
36–45 years	155 (22.5%)
>45 years	119 (17.2%)
Sex	
Male	296 (42.9%)
Female	394 (57.1%)
Nationality	
Saudi	686 (99.4%)
Non-Saudi	4 (0.6%)
BMI level	
<40	146 (21.1%)
40-49.9	373 (54.1%)
≥50	171 (24.8%)
Use of PPI	
Yes	12 (01.7%)
No	678 (98.3%)

**Figure 1:**
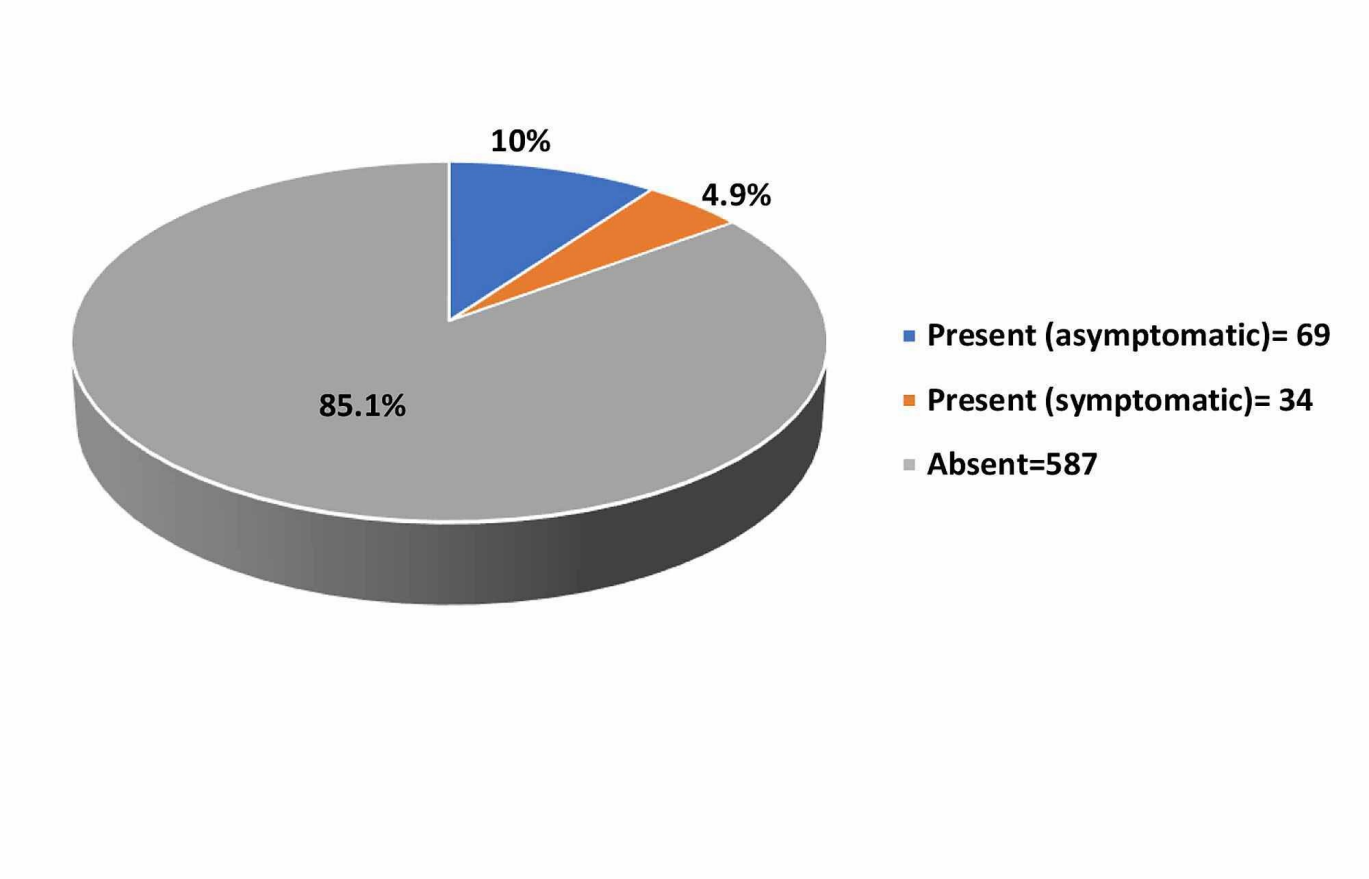
Prevalence of HH among obese patients HH, hiatal hernia

Figure 1 shows the prevalence of HH in obese patients. The prevalence of HH among obese patients was 14.9% (103), of whom 67% (69) were asymptomatic.

In Table 2, the results show the prevalence of HH among different demographic variables. Patients who were < 35 years old (45.63%) were found to have asymptomatic HH. Most patients who had high BMI were found to experience some symptoms related to HH.

**Table 2:** Relationship between hiatal hernia among the basic demographic and associated diseases of obese patients (n=690) §The p-value was calculated using the chi-square test. **Significant at p<0.05. *Variable with multiple responses. BMI, body mass index; PPI, proton-pump inhibitors; DM, diabetes mellitus; HTN, hypertension

Factor	Hiatal Hernia			X^2^	p-Value^§^
	Present, N (%) (n=103)		Absent, N (%) (n=587)		
	Asymptomatic	Symptomatic			
Age group					
≤35 years	47 (45.6%)	19 (18.5%)	350 (59.6%)	0.726	0.394
>35 years	20 (19.4%)	17 (16.5%)	237 (40.4%)		
Sex					
Male	32 (31%)	18 (17.5%)	246 (41.9%)	1.575	0.209
Female	35 (34%)	18 (17.5%)	341 (58.1%)		
Nationality					
Saudi	67 (65.05%)	36 (34.95%)	583 (99.3%)	0.706	0.401
Non-Saudi	0 (0%)	0 (0%)	4 (0.7%)		
BMI level					
<40	17 (16.5%)	7 (6.8%)	122 (20.8%)	2.126	0.345
≥40	30 (29.1%)	18 (17.5%)	324 (55.2%)		
≥50	20 (19.4%)	11 (10.7%)	141 (24.0%)		
Use of PPI					
Yes	3 (2.9%)	2 (2%)	7 (1.2%)	6.876	0.009**
No	64 (62.1%)	33 (33%)	580 (98.8%)		
Chronic diseases*					
Asthma	3 (2.91%)	1 (0.97%)	44 (7.5%)	1.766	0.184
DM	5 (4.85%)	4 (3.9%)	90 (15.3%)	3.101	0.078
HTN	3 (2.91%)	1 (0.97%)	44 (7.5%)	1.766	0.184
Hypothyroidism	6 (5.82%)	1 (0,97%)	56 (9.5%)	0.795	0.373

**Figure 2:**
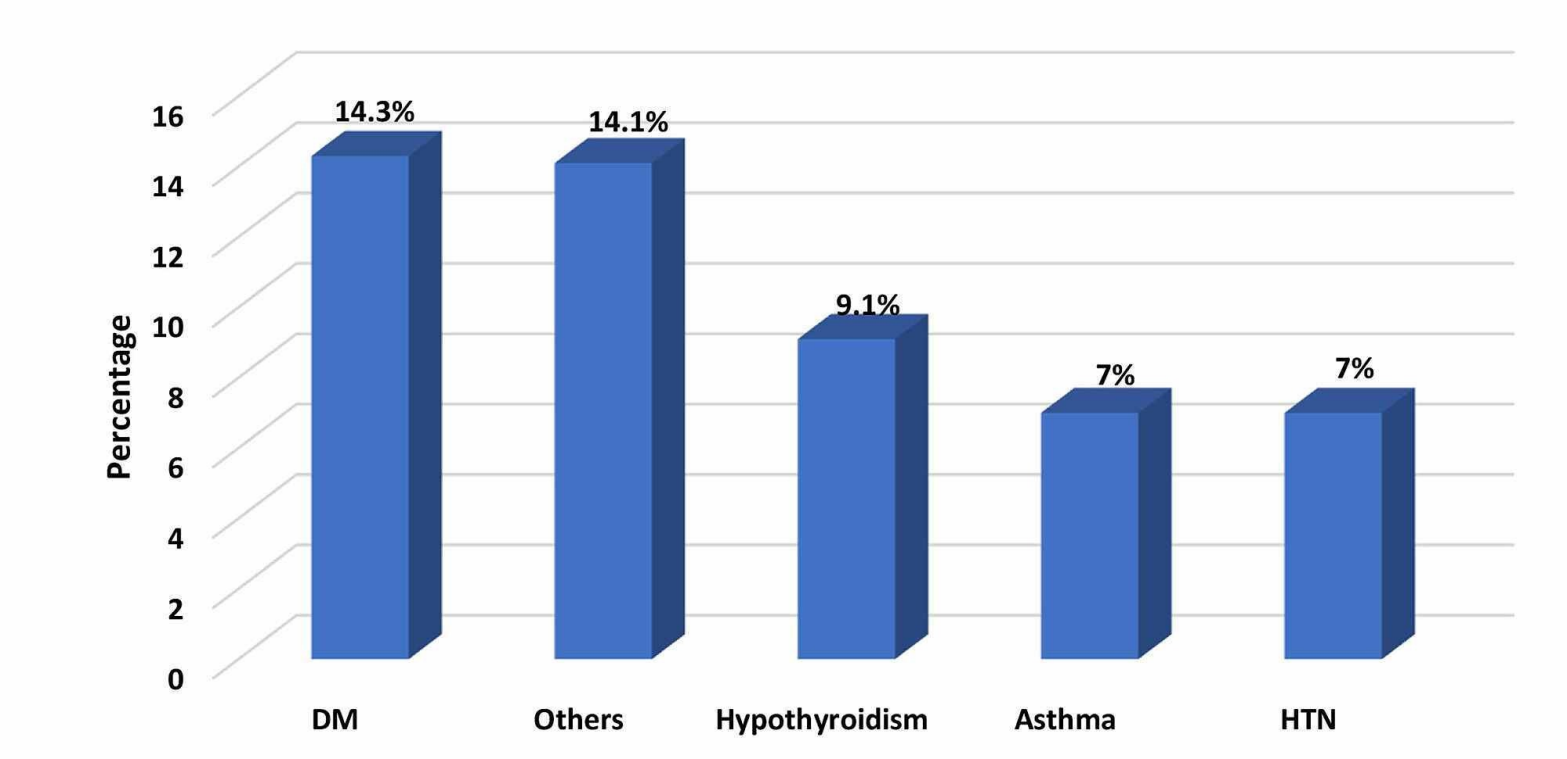
Chronic diseases in obese patients

Figure 2 presents chronic diseases associated with obese patients. The most frequently cited chronic disease was diabetes mellitus (14.3%) followed by hypothyroidism (9.1%), hypertension (7%), and asthma (7%).

In Table 3, chi-square tests were performed to determine the symptoms related to HH. The results showed that abdominal pain (X2=3.885; p=0.049), shortness of breath (X2=8.057; p=0.005), vomiting (X2=4.302; p=0.038), and nausea (X2=4.090; p=0.043) were significantly associated with the presence of HH.

**Table 3:** Symptoms related to hiatal hernia §The p-value was calculated using the chi-square test. *Variable with multiple responses. **Significant at p<0.05.

Factor	Hiatal Hernia		X^2^	p-Value^§^
	Present, N (%) (n=103)	Absent, N (%) (n=587)		
Symptoms of hiatal hernia*				
Heartburn	14 (13.6%)	68 (11.6%)	0.337	0.561
Difficulty in swallowing	7 (6.8%)	21 (3.6%)	2.332	0.127
Chest pain	7 (6.8%)	21 (3.6%)	2.332	0.127
Abdominal pain	23 (22.3%)	86 (14.7%)	3.885	0.049**
Shortness of breath	3 (2.9%)	2 (0.3%)	8.057	0.005**
Vomiting	23 (22.3%)	84 (14.3%)	4.302	0.038**
Nausea	23 (22.3%)	85 (14.5%)	4.090	0.043**
Others	2 (1.9%)	2 (0.3%)	3.897	0.048**

Multivariate regression estimates (Table 4) showed an independent significant factor associated with HH. PPI use was found to decrease the symptoms associated with HH (adjusted OR [AOR]=0.237; 95% CI=0.074-0.760; p=0.023). Patients with vomiting were nearly two-fold more likely to have HH (AOR=1.722; 95% CI=1.025-2.890; p=0.040), while patients with nausea had a 1.6-fold higher risk (AOR=1.698; 95% CI=1.012-2.849; p=0.045).

**Table 4:** Multivariate regression analysis to detect the independent significant predictor associated with hiatal hernia (n=690) **Significant at p<0.05. AOR, adjusted odds ratio; CI, confidence interval; PPI, proton-pump inhibitors

Factor	AOR	95% CI	p-Value
Use of PPI			
Yes	0.237	0.074–0.760	0.023**
No	Ref		
Abdominal pain			
Yes	1.587	0.920–2.739	0.097
No	Ref		
Shortness of breath			
Yes	4.987	0.739–33.664	0.099
No	Ref		
Vomiting			
Yes	1.722	1.025–2.890	0.040**
No	Ref		
Nausea			
Yes	1.698	1.012–2.849	0.045**
No	Ref		
Other symptoms			
Yes	6.666	0.925–48.049	0.060
No	Ref		

## Discussion

HH is a stomach disorder that involves herniation of the abdominal cavity. In the United States from 2003 to 2006, HH was the primary and secondary cause of hospitalization in 142 of 10,000 inpatients [5]. However, the exact prevalence of HH is difficult to ascertain owing to the inherent diagnostic criteria. In a study performed in Romania, preoperative investigations such as upper endoscopy and barium swallow X-ray studies are less sensitivity in some cases, which explains that almost half (43.37%) of the patients with HHs were discovered intraoperatively [7]. In this study, we sought to determine the prevalence of asymptomatic HH among obese patients and to evaluate whether it is associated with BMI. The prevalence of asymptomatic HH in this study was low (14.9%). Several studies have documented the prevalence of HH among obese patients or patients with GI problems, ranging from 9.3% to 37% [2,8-12]. Che et al. [1] reported the highest prevalence of HH (37%), while Hyun et al. [11] reported a very low prevalence (9.3%). The prevalence of HH in this study was consistent with that reported by Petersen et al. [12], who reported a prevalence of 17% among patients with gastroesophageal reflux symptoms.

Age and obesity are the most common risk factors for HH [12-14]. Compared to people with normal body weight, overweight or obese people have a progressive increase in intra-abdominal pressure, which leads to herniation [15]. Another study found that the presence of HH was significantly associated with excessive body weight, and the probability of HH increased with each level of BMI [16]. This has been validated in a meta-analysis conducted by Menon and Trudgill [17], who observed that the odds ratio for HH in people with a BMI greater than 25 was 1.93 (95% CI: 1.10-3.39), with the risk increasing as the BMI increased. However, in our study, we failed to prove a correlation between BMI and HH (X2=2.126; p=0.345); age was also not a significant predictor, in contrast to previous results.

Univariate analysis determined that the most frequently reported symptoms of HH were abdominal pain, shortness of breath, vomiting, nausea, and others. However, after performing multivariate regression estimates, we found that the use of PPI medication was found to decrease the symptoms associated with HH. Other reports studied sex and HH and found that males were more frequently diagnosed with HH than females [8,17]. In the present study, the relationship between sex and HH did not differ significantly, in contrast to previous results.

Moreover, this study suggests that no specific symptoms are associated with HH. Symptoms related to gastroesophageal reflux, including heartburn, regurgitation, and dysphagia, were linked to HH due to the occurrence of hernia [18]. In our study, the most common symptoms related to HH were nausea (15.7%) followed by vomiting and abdominal pain (each 15.5%), and shortness of breath was the least common symptom (0.7%), which is not consistent with previous findings.

## Conclusions

Asymptomatic HH among obese patients is uncommon in Saudi Arabia. The use of PPI medications was found to decrease the symptoms associated with HH, while symptoms such as vomiting and nausea were the most frequently reported in those with HH. Conversely, there was no evidence linking BMI to the development of HH. More research is needed to validate the prevalence of asymptomatic HH in obese patients in our region.
